# Droplet Digital PCR for absolute quantification and determination of proviral load of HTLV-1 and 2

**DOI:** 10.1186/1742-4690-12-S1-P71

**Published:** 2015-08-28

**Authors:** Sara Thulin Hedberg, Lorraine Eriksson, Kerstin Malm, Paula Mölling, Martin Sundqvist, Sören Andersson

**Affiliations:** 1 National Reference Laboratory for HTLV, Department of Laboratory Medicine, Faculty of Medicine and Health, Örebro University Hospital, Örebro, Sweden

## 

HTLV proviral load in PBMCs is one of the few laboratory parameters used for clinical follow up and assessment of development of symptomatic disease associated with HTLV infection. High levels of proviral load are associated with symptomatic disease (e.g. ATLL, HAM/TSP) and occurance of higher levels among asymptomatic individuals has been suggested prognostic for development of symptomatic HTLV disease. Commonly, standard PCR methods including standard curves have been used. Droplet Digital PCR (ddPCR) is a new method that enables absolute quantification of DNA and RNA without the use of a standard curve. In ddPCR, the PCR sample is partitioned into 20 0 nanoliter-sized droplets prior to PCR and based on the number of positive droplets and Poisson algorithms the concentration of the sample can be determined. We have established and evaluated an assay for identification, quantification and determination of PVL of HTLV-1 and 2 in PBMC using ddPCR. For determination of PVL, the human gene RPP30 was quantified and used as reference [1]. The samples were extracted using the QIAamp DNA Blood Mini kit on the QIAcube (Qiagen) prior to ddPCR. Sixty HTLV-1 – positive (PVL 0.01%-59.8%) and 12 HTLV-2 – positive samples (PVL 0.02%-11.3%) previously analyzed with real-time PCR at Imperial College, St Mary's Hospital, London (Prof Graham Taylor) as well as 20 negative samples and dilution series of both HTLV-1 and 2 were examined. The results of ddPCR were in concordance with real-time-PCR, however a few weak positive samples were only detected by one of the methods, see Figure 1. The detection levels of the ddPCR were determined to 1-2 copies per PCR reaction for both HTLV-1 and 2. Both the intra- and inter-assay variability of ddPCR was low, 0.97-3.34% and 1.84-6.13% respectively. In conclusion, ddPCR allows for reliable and sensitive determination of PVL for both HTLV-1 and 2.

